# Lactate, Carbohydrate Restriction, and Cancer Metabolism

**DOI:** 10.1159/mpp/adpag001

**Published:** 2026-08-03

**Authors:** Yunus A. Luqmani

**Affiliations:** Department of Pharmaceutical Sciences, College of Pharmacy, Kuwait University, Kuwait City, Kuwait

**Keywords:** Lactate, Carbohydrate restriction, Ketogenic diet, Cancer metabolism, Metabolic reprogramming, Tumour microenvironment, Warburg effect, Immune suppression

## Abstract

Carbohydrate restriction has been proposed as an adjunctive strategy in cancer, largely because many tumours show high glucose uptake. Clinical evidence for benefit remains limited, mixed, and often overstated. Lactate is now recognised as more than a metabolic by-product: it influences redox balance, tumour-stromal signalling, immune suppression, angiogenesis, epigenetic regulation, and metastatic behaviour. This review considers carbohydrate-restrictive diets from a lactate-centred perspective. The stronger mechanistic argument is not simple glucose deprivation, but the possibility that lower carbohydrate availability may reduce glucose-to-lactate flux and disrupt lactate-dependent tumour ecosystems in selected contexts. It discusses where this idea is biologically plausible, where claims run beyond the data, and how dietary approaches compare with direct lactate-targeted strategies. To ground the clinical discussion, registered interventional trials of caloric and/or carbohydrate manipulation in cancer were reviewed; they show an active but early-stage field dominated by small, heterogeneous studies and few direct lactate-related endpoints. Future trials should use biomarker-guided tumour selection, direct measures of tumour lactate flux, immune and microenvironmental endpoints, and explicit safeguards for weight, muscle mass, treatment tolerance, and quality of life. At present, carbohydrate restriction should remain investigational and be tested only in well-designed clinical studies with clear nutritional safeguards.

## Plain Language Summary

Lactate is a normal and important metabolite in healthy tissues often produced as a “temporary stop gap” product when oxygen supply is limiting. It participates in energy transfer, redox balance, and communication particularly between the muscle and liver. In cancer, lactate can become much more prominent and potentially pivotal. Many tumours use glucose at high rates and convert substantial amounts of it into lactate, even when oxygen is available, exporting it into the extracellular space. This lactate-rich environment can promote tumour survival, immune suppression, angiogenesis, stromal remodelling, invasion, and metastatic behaviour. This provides a strong biological rationale for considering dietary carbohydrate restriction as an adjunct to conventional cancer treatment. However, the aim should not simply be to “starve” cancer cells of sugar per se since blood glucose is tightly regulated and tumours can adapt by using alternative fuels. The more persuasive argument is that reducing carbohydrate availability may lower glucose-driven lactate production and weaken lactate-dependent tumour processes. Clinical evidence, however, remains limited, and carbohydrate restriction should not be presented as a proven anticancer treatment. Even so, the lack of large definitive trials does not negate the mechanistic rationale. Evidence from cancer metabolism, lactate biology, immune regulation, and tumour adaptation supports serious investigation of carbohydrate restriction as a complementary metabolic strategy, particularly in tumours with high glucose uptake, hypoxia, lactate production, or glycolytic dependence. Any such approach must be used alongside, not instead of, standard cancer treatments such as surgery, radiotherapy, chemotherapy, targeted therapy, or immunotherapy. It also requires medical/nursing supervision because some cancer patients may be vulnerable to weight loss, muscle loss, fatigue, nutritional deficiency, and reduced treatment tolerance. Overall, dietary carbohydrate restriction is not yet proven, but it is biologically plausible, clinically relevant and deserving of structured testing as an adjunctive strategy in selected cancer settings.

**Highlights of the Study**

- Lactate provides a stronger mechanistic focus than simple glucose starvation
- Carbohydrate restriction may, in selected tumour settings, influence lactate production, lactate flux, and lactate-dependent signalling; this requires direct clinical testing
- Current clinical trials remain small, heterogeneous, and mainly focused on feasibility, safety, and exploratory endpoints
- Future studies should measure tumour lactate flux, immune effects, and nutritional safety with larger cohorts.

## Introduction

Cancer has often been described as a genetic disease, driven by somatic mutation, chromosomal instability, and dysregulated signalling pathways [1]. This view has been productive. It has supported molecular classification, biomarker-guided treatment and the development of targeted therapies and immunotherapies that help selected groups of patients [2]. But genotype is not the whole story. It does not, by itself, explain tumour adaptability, persistence after therapy, or remodelling of the tumour microenvironment. Cancer is now better understood as a disease of interacting biological systems. Within that wider view, metabolic reprogramming is not a side issue. It is one of the processes that helps malignant cells survive after normal controls over growth, nutrient use and tissue function have been lost [3]. Deregulated cellular energetics is now accepted as a hallmark of cancer [1]. Metabolic adaptation supports proliferation, but it also contributes to invasion, angiogenesis, immune escape, and resistance to therapy [4].

Tumour metabolism is shaped by several pressures at once: oncogenic signalling, tissue of origin, perfusion, hypoxia, systemic hormones, nutrient availability and two-way interactions with stromal and immune cells [5]. The result is metabolic heterogeneity across both space and time. That heterogeneity can affect treatment response and relapse [6]. Interventions that alter systemic metabolism therefore deserve careful study, both as possible adjuncts and as tools for probing tumour metabolic dependencies in humans [3]. Dietary strategies have become part of this discussion. Traditionally, diet in oncology was mainly supportive: maintain nutritional status, reduce weight loss, and manage cachexia [7, 8]. More recently, diet has also been considered as a possible metabolic intervention, able in principle to alter systemic hormones and tumour fuel use [9]. Carbohydrate-restrictive diets (CRDs), including very-low carbohydrate and ketogenic regimens, have attracted particular interest. The reason is straightforward: many tumours show high glucose uptake and sustained conversion of glucose to lactate, even when oxygen is available [10, 11].

A common public and clinical message is that reducing carbohydrate will “starve” cancer cells of glucose, while normal tissues simply switch to fatty acids and ketone bodies [12]. The idea is attractive, but too simple. Glucose remains essential for normal physiology. Lower dietary carbohydrate does not mean systemic glucose deprivation, and high glucose uptake does not prove that a tumour is absolutely glucose-dependent [4, 10]. A more useful focus may be lactate. Lactate is a major product of tumour glucose metabolism, but it is also an active regulator of the tumour microenvironment rather than a passive waste product [13–16]. Historically, lactate was often treated as an end product, sometimes even as waste, despite early recognition through the Cori cycle that lactate moves between the muscle and liver. Its active role in cancer progression has received sustained attention only relatively recently. This review evaluates carbohydrate restriction through that lactate-centred perspective. The most coherent rationale for CRDs is not “glucose starvation” as such. It is a possibility, still unproven clinically, that carbohydrate restriction could reduce glucose-to-lactate flux in selected tumours and thereby disrupt lactate-dependent tumour ecosystems [16–19]. This review first summarises lactate biology in cancer, then considers how carbohydrate restriction might influence lactate production and downstream effects, and where the current evidence remains plausible but clinically unproven [12, 20]. It also discusses host risk, tumour adaptation and how dietary strategies compare with more direct lactate-targeted approaches [21].

## Glucose, Lactate, and Metabolic Selection in Cancer

The conversion of glucose to lactate despite the presence of oxygen has been recognised for almost a century as a feature of many cancers [22]. Early explanations suggested defective mitochondrial respiration. That view may now be considered as too narrow. Many tumours retain functioning mitochondria and substantial oxidative capacity [2]. Aerobic glycolysis is better understood as a regulated phenotype that can be advantageous when the microenvironment is unstable [13]. Tumours often contain steep oxygen gradients, fluctuating perfusion and uneven nutrient access [23]. Glycolysis can generate ATP quickly under hypoxia, sustain redox balance and support biosynthesis [5]. Regeneration of NAD^+^ by lactate dehydrogenase (LDH) allows glycolysis to continue when mitochondrial oxidative capacity is saturated or oxygen-limited [11]. Oncogenic programmes, including Phosphoinositide 3-kinase (PI3K)-Protein Kinase B (AKT), MYC proto-oncogene, bHLH transcription factor (MYC) and hypoxia-inducible factor-driven pathways, reinforce this pattern by increasing glucose transport and glycolytic enzyme expression [24].

In oxygenated differentiated cells, glucose-derived pyruvate is commonly oxidised in mitochondria through the tricarboxylic-acid cycle and oxidative phosphorylation. Lactate production in normal tissues is nevertheless physiologically important during hypoxia, intense glycolytic activity, redox stress, and inter-organ carbon transfer. The Warburg phenotype differs not because lactate is unique to cancer, but because many malignant cells sustain high glycolytic flux and lactate export even when oxygen is available. This reflects a combination of proliferative demand, redox control, biosynthetic needs, hypoxia gradients, and oncogenic signalling. Insulin and insulin-like growth factor (IGF) pathways can increase glucose uptake and glycolytic activity through PI3K-AKT-mechanistic target of rapamycin (mTOR) and related nutrient-sensing pathways, but tumours vary in their dependence on systemic insulin/IGF because many also acquire autonomous activation of the same downstream programmes [11, 24–27].

### Lactate Accumulation and Tumour Fitness

Lactate concentrations in solid tumours can reportedly reach 10–40 mmol/L, depending on how and where they are measured, and are often much higher than in normal tissues [28, 29]. This reflects coordinated upregulation of glucose transporters, glycolytic enzymes, LDH isoforms and monocarboxylate transporters [30]. Lactate is not merely a temporary carbon store in oxygen deprived cells. It can support the integrity of both cancer cells and the wider tumour ecosystem [31]. In heterogeneous tumours, lactate allows metabolic symbiosis. Hypoxic glycolytic cells export lactate; more oxidative tumour cells and stromal cells may import and oxidise it as a carbon source [17]. This division of labour uses spatial heterogeneity to reduce competition for glucose in better-perfused regions [32]. Lactate export also helps regulate intracellular pH and preserves the glycolytic-redox state needed for biosynthesis and stress tolerance [33].

### Lactate as a Multifunctional Effector

Lactate acts as both substrate and as a signalling molecule [34]. In the tumour microenvironment, high lactate can suppress immunity by inhibiting effector T-cell and natural killer-cell function, partly through effects on glycolysis and cytokine output [35, 36]. It can favour immunoregulatory phenotypes, including regulatory T cells and suppressive myeloid populations, and may push macrophages towards tumour supportive states [37]. Lactate also promotes angiogenesis and stromal activation [14]. It may stimulate endothelial cell migration, alter vascular remodelling and contribute to extracellular-matrix changes that support invasion [38, 39]. At the cellular level, lactate is linked to redox control through NAD^+^/NADH and can influence pathways sensitive to redox state [11]. It has also been reported to affect transcription through epigenetic mechanisms, including histone lactylation [40]. Taken together, these findings make lactate a plausible integrative driver of malignant progression, not simply a metabolic product [16, 31]. These lactate-driven metabolic and microenvironmental interactions are summarised schematically in Figure 1.

**Fig. 1.**
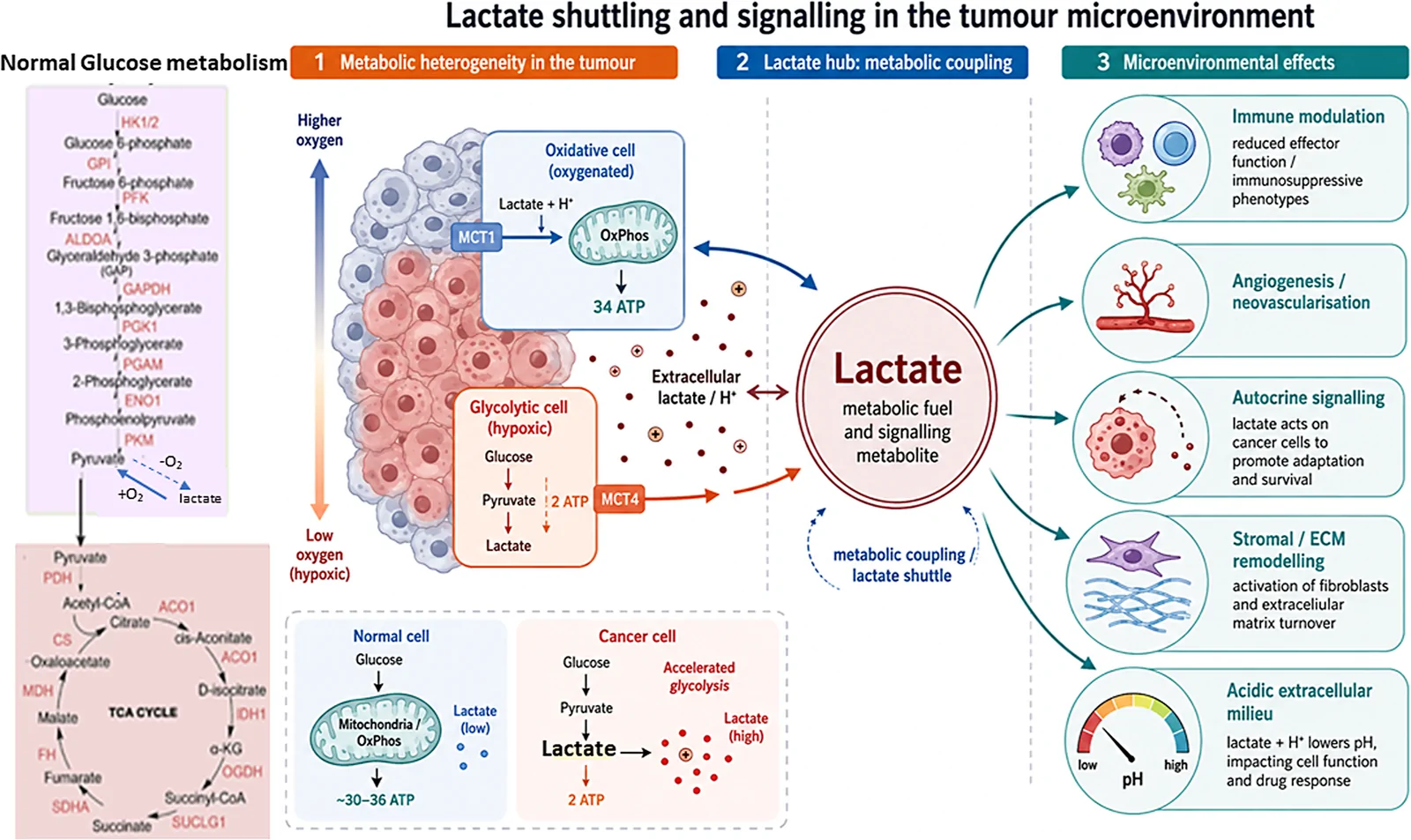
Altered glucose metabolism and lactate-driven signalling in cancer cells; interaction with extracellular environment. Schematic representation of glucose metabolism, lactate production, lactate exchange and downstream signalling in cancer. In normal cells, glucose is metabolised through glycolysis to pyruvate, which usually enters mitochondrial oxidation through the tricarboxylic-acid cycle and oxidative phosphorylation. In tumours, uneven oxygen availability creates metabolically distinct cell populations. Hypoxic glycolytic cancer cells preferentially convert glucose to lactate, generating limited ATP and exporting lactate and H+ through MCT4. More oxygenated oxidative cancer cells can import lactate through MCT1 and use it as a substrate for oxidative phosphorylation. This illustrates metabolic coupling and lactate shuttling within the tumour. Accumulated extracellular lactate also acts as a signalling metabolite. It contributes to immune modulation, angiogenesis and neovascularisation, autocrine cancer-cell adaptation, stromal and extracellular-matrix remodelling, and extracellular acidification. Together, these processes position lactate as both a metabolic fuel and a regulator of tumour-microenvironment interactions that may promote dissemination. ECM, extracellular matrix; MCT, monocarboxylate transporter; OxPhos, oxidative phosphorylation; TCA, tricarboxylic acid. Left panel is modified from part of Figure 1. In Chen et al. [41], reproduced under the Creative Commons Attribution License.

Lactate can also act through receptor-linked and intracellular signalling mechanisms. The hydroxycarboxylic-acid receptor HCAR1/GPR81 is activated by lactate and has been implicated in cancer-cell survival, angiogenic signalling and immune modulation in some models. Lactate transport through monocarboxylate transporter (MCT) 1 and MCT4, with cluster of differentiation 147 as an accessory protein, is not merely a disposal system; it shapes intracellular pH, redox state, substrate exchange, and metabolic coupling. Lactate has also been reported to stabilise N-Myc Downstream-Regulated Gene (NDRG) 3 under hypoxia and to influence transcriptional programmes through histone lactylation. These mechanisms remain context-dependent, but they help explain how chronically high lactate can benefit malignant tissue by supporting immune evasion, angiogenesis, stromal remodelling, invasion, and adaptation to stress, while the same metabolite continues to have normal physiological roles in healthy tissues [40, 42–48].

### Why Lactate Is a Relevant Target

Glucose is indispensable and tightly regulated in humans and other animals [25]. A low-carbohydrate diet can reduce postprandial glucose and insulin, but circulating glucose remains available [27]. Tumours may also adapt to lower glucose availability by improving uptake efficiency or by turning to glutamine, fatty acids, or lactate itself [32]. Lactate provides a distinct metabolic perspective. In many tumours, it is pathologically amplified and is linked to several tumour-promoting processes [13, 16]. The central dietary question is therefore not simply whether a diet produces ketosis, but whether carbohydrate restriction can reduce intratumoural lactate flux in selected contexts and disturb lactate-dependent tumour ecology; this remains a hypothesis requiring direct clinical testing [19, 49].

### Lactate Production, Circulating Lactate, and Lactate Use

It is important to distinguish three related but different issues. Firstly, tumours may produce lactate from glucose through glycolysis and export it through monocarboxylate transporters. Secondly, lactate can be exchanged within the tumour microenvironment: hypoxic glycolytic cells can export lactate, whereas more oxygenated tumour cells or stromal cells may import and oxidise it. Thirdly, lactate is also a normal circulating metabolite produced by host tissues and used in inter-organ metabolism, including the liver, kidney, heart, brain and skeletal muscle. Evidence that glucose is a major precursor of circulating lactate in normal physiology does not by itself define lactate flux within malignant tissue. Conversely, high tumour lactate does not prove that systemic carbohydrate restriction will reduce tumour lactate. A lactate-centred dietary hypothesis therefore requires direct tumour or tumour-proximal measures of lactate production, export, and utilisation [13, 15, 17, 32, 50].

## Mechanistic Rationale for Carbohydrate Restriction

CRDs can reduce circulating glucose levels and insulin secretion in some clinical settings. In theory, a lower glucose, lower insulin systemic environment could reduce glycolytic throughput in tumour cells that remain responsive to systemic cues. That, in turn, might reduce lactate production and export [9, 51–53]. If this occurred, downstream effects could include less extracellular lactate and acidity, less lactate-mediated immune suppression, weaker metabolic symbiosis, altered angiogenic signalling, and changes in redox balance during stress [17, 37, 38, 49]. These steps should not be assumed. Tumour glucose uptake is often driven by oncogenic signalling and may be only partly linked to circulating glucose [3]. Some tumours may use circulating lactate produced by host tissues, or switch towards alternative substrates when nutrients are perturbed [15, 50]. Mechanistic studies therefore need direct measurements of lactate flux in vivo – production, export and use – together with lactate-dependent pathways. Ketosis and dietary composition alone are therefore inadequate proxies for these metabolic effects [54, 55].

These relationships should be interpreted as a testable chain, not as an established clinical sequence. Dietary carbohydrate intake can influence postprandial glucose and insulin; glucose and insulin can influence glycolytic flux in responsive tissues; and glycolysis can generate lactate. However, blood glucose, tumour glucose uptake, and intratumoural lactate flux are not interchangeable measures. Oncogenic signalling, hypoxia, perfusion, substrate competition and metabolic adaptation may all weaken or reshape the connection between diet and tumour lactate [10, 24, 26, 27].

### Insulin/IGF Signalling and Nutrient-Sensing Pathways

Carbohydrate restriction often lowers insulin and can reduce insulin-like growth factor signalling, depending on total energy balance and protein composition [9]. These changes may affect nutrient-sensing pathways such as PI3K-AKT-mTOR and 5′ AMP-activated protein kinase, which regulate cell growth, metabolism, and survival [24, 27]. Because abnormal PI3K signalling is common in cancer, reduced systemic growth signalling has been proposed as one way CRDs might slow tumour growth or increase sensitivity to cytotoxic therapy [27]. A difficulty is that tumours vary in their responsiveness to systemic insulin and IGF signals. Many have ligand-independent activation of growth pathways, or autocrine loops that blunt the effect of systemic change [2]. Protein intake also influences IGF-1 levels. Without protein standardisation, it is difficult to isolate carbohydrate-specific effects [26]. Claims that carbohydrate restriction acts through insulin-IGF pathways are plausible, but often confounded by calorie and protein effects [56]. A lactate-rich microenvironment can suppress immune effector function, promote immunoregulatory populations and may be linked to reduced treatment efficacy in some settings [16, 35, 36, 49, 57].

If caloric restriction mimetics (CRMs/CRDs) reduce lactate accumulation, they might relieve parts of this immune suppression or improve response to immune checkpoint blockade in defined contexts [58]. Lactate-driven angiogenic and stromal programmes might also be affected by reduced lactate flux, with possible effects on perfusion, hypoxia, and the invasive niche [38]. These microenvironmental claims remain unproven hypotheses. Hypoxia, cytokine gradients, nutrient competition, extracellular-matrix composition, and lactate are closely intertwined. In advanced disease, systemic dietary change may not alter these variables enough to matter [23, 31]. Strong clinical inference will require paired measures of tumour lactate, immune metabolism and spatial immune architecture, not just dietary adherence or ketone levels [54, 55, 59].

This distinction is important because serum glucose, dietary carbohydrate intake, Fluorodeoxyglucose – Positron Emission Tomography (FDG-PET) avidity, insulin/IGF signalling and tumour lactate flux are related but non-interchangeable variables. FDG-PET demonstrates avid glucose uptake in many tumours, but it does not by itself prove that dietary carbohydrate reduction will suppress intratumoural lactate flux or improve outcome [25, 60].

### Ketone Bodies: Fuel versus Signal and Limits of Selectivity

Ketogenic diets increase circulating ketone bodies, which many normal tissues can utilise [61]. One common claim is that ketones feed normal tissues while cancer cells are starved [62]. The evidence does not support this as a general rule. Some tumours, including glioblastomas, express ketolytic enzymes and can oxidise ketone bodies under certain conditions [63]. Ketones are also signalling metabolites, with complex effects on stress responses and gene regulation [64]. The more careful mechanistic position is that ketosis is an accompanying systemic state. The key upstream question is whether carbohydrate restriction changes lactate flux and lactate-dependent biology [18].

## Appraising the Evidence: Benefits, Limitations, and Boundary Conditions

### Where Plausibility Is Highest: Metabolic Constraints and Therapy Context

If carbohydrate restriction is to be tested as an adjunctive strategy, tumour selection matters greatly [56]. The most plausible candidates are tumours with high glycolytic reliance, substantial lactate production, hypoxic signatures, high LDH or MCT4 expression, and limited metabolic flexibility [29, 31, 33]. High-grade gliomas are often discussed in this context. They commonly show high glucose uptake and prominent lactate production, while normal brain tissue can use ketone bodies under physiological conditions [61, 65, 66]. Even so, “metabolic constraint” is not an on-off state. Tumours are genetically heterogeneous, and different cell populations may vary in ketolysis, fatty-acid oxidation, glutamine dependence, and lactate uptake [10, 24]. Biomarker-driven selection is therefore important [5]. Negative results in unselected groups should not automatically disprove lactate-modulation hypotheses. Conversely, signals in small cohorts should be read cautiously until replicated in larger studies with endpoints robust enough to interpret [67, 68]. Radiotherapy and some chemotherapy regimens work partly through oxidative stress and DNA damage [69–71].

If carbohydrate restriction or ketosis weakens tumour redox buffering in selected contexts, radio sensitisation is biologically plausible [72, 73]. From a lactate-centred view, lower glycolytic throughput could reduce pentose phosphate pathway flux and impair redox buffering in tumours already close to oxidative limits [73]. Similarly, if lactate suppression improves immune effector function, there is a rationale for combining metabolic interventions with immunotherapy [58]. These ideas are promising, but still incompletely tested in humans [72, 74].

Lactate dependence should not be treated as universal. Tumours differ by tissue of origin, driver mutations, vascularity, hypoxia, stromal composition, and treatment history. Even within a single tumour, hypoxic glycolytic regions, better oxygenated oxidative regions, stromal cells, and immune cells may use different fuels. Some cancer-cell populations primarily export lactate, whereas others may import and oxidise lactate or switch towards glutamine or fatty-acid metabolism. This heterogeneity means that histological diagnosis alone is unlikely to identify patients most likely to benefit from carbohydrate-restrictive or lactate-modulating strategies. Future studies should therefore select or stratify patients using metabolic markers such as FDG uptake, LDH expression, MCT4 expression, hypoxia signatures and, where feasible, direct lactate imaging, or metabolic profiling [6, 13, 17, 24, 75].

### Universality and Fuel-Deprivation Claims

The most common overstatement is that cancers are generally glucose-dependent and can be starved by lowering dietary carbohydrate [12]. High glucose uptake does not prove obligate dependence. Many tumours can oxidise lactate and fatty acids, or increase amino acid use when glucose is constrained [24]. In humans, lactate is actively exchanged between tissues, most notably between muscle and liver, and can become a major circulating fuel during physiological stress [50]. Some tumours may therefore use lactate even when glucose availability is altered. Broad claims that carbohydrate restriction deprives tumours of fuel are biologically and clinically misleading [12].

### Calorie/Protein Confounding

A second overstatement involves calorie and protein effects. Ketogenic interventions often reduce appetite and total caloric intake, and this alone can influence metabolic and growth-signalling endpoints [53, 76]. Protein intake can also alter IGF-axis biology, which further complicates interpretation if protein is not standardised [26]. Good trials therefore need isocaloric designs, protein standardisation, and objective adherence monitoring where the question is specifically about macronutrient composition rather than energy deficit [56, 77].

### Translation from Preclinical Models

Animal studies often use extreme diets, controlled environments, and relatively homogeneous tumour models. These conditions can exaggerate effect sizes and limit generalisability [74]. Human cancers are more variable, and they occur in people with different comorbidities, treatment regimens and nutritional reserves [5]. Preclinical work is useful for generating hypotheses and finding biomarkers. It cannot replace rigorous clinical trials [68]. Claims of efficacy based only on preclinical models should therefore be treated with caution [77, 78].

## Biological Risks and Trade-Offs

### Host Risks: Weight Loss, Sarcopenia, and Treatment Tolerance

Cancer patients are often already at risk of weight loss, sarcopenia, and cachexia, all of which are linked to poorer outcomes and reduced tolerance of systemic therapy [8, 79]. Carbohydrate restriction could worsen these risks if it reduces calorie intake, becomes burdensome, or leads to inadequate protein or micronutrient intake [80, 81]. Some patients also develop gastrointestinal symptoms, fatigue or metabolic disturbances that make adherence difficult [20, 53]. The risk is likely to be highest in patients receiving intensive multimodal therapy, where even small losses of functional reserve can affect dose intensity and outcomes [82]. These risks are not theoretical. In many cancer populations, reduced appetite, taste change, mucositis, nausea, diarrhoea, fatigue and treatment-related catabolism already make adequate intake difficult. A restrictive diet may unintentionally produce calorie deficit when weight loss is not the intended goal. Protein inadequacy, micronutrient insufficiency and loss of skeletal muscle can reduce physical function and may compromise treatment tolerance. Trials should therefore measure not only body weight but also body composition, functional status, dietary intake, patient-reported burden, treatment delays, dose reductions, and hospitalisations. Protocols should include prespecified rescue criteria for clinically meaningful weight loss, muscle loss, or functional decline [80–84].

### Monitoring, Stopping Rules, and Support

Clinical implementation would need nutritional monitoring, body-composition assessment, clear stopping rules and dietetic support [80, 83]. Feasibility and equity also matter. Intensive dietary protocols depend on resources, health literacy, food access, household support, and patient capacity. Access to these resources and capabilities varies substantially between patients and populations [7].

### Tumour Evolutionary Trade-Offs and Metabolic Escape

Metabolic interventions impose selection pressures [29]. Tumours may respond to lower carbohydrate availability by increasing lactate uptake, enhancing fatty-acid oxidation, shifting towards glutamine dependence or relying more heavily on stromal support [10, 23]. Such adaptation could preserve growth and, in some contexts, select for more aggressive phenotypes [16, 23]. A lactate-centred approach therefore has to look for compensatory pathways over time, using longitudinal biomarkers to detect metabolic escape [19]. If lactate flux is not reduced, or if immune suppression persists, the mechanistic premise has not been achieved even when ketosis has been achieved [58].

## Dietary Approaches versus Direct Lactate-Targeted Strategies

Dietary carbohydrate restriction is a systemic intervention, and its effects on tumour metabolism are variable [78]. Direct approaches – such as inhibition of lactate dehydrogenase, blockade of lactate transport through MCT inhibition, modulation of pH regulation, or interference with lactate signalling – are more mechanistically specific. They can be titrated, combined and assessed with pharmacodynamic biomarkers [16, 21, 85–89]. Dietary interventions may still have a place, but mainly as adjuncts or experimental perturbations, and only when combined with therapy and evaluated with mechanistic endpoints [77].

Other metabolic-targeting approaches also deserve mention, although they are mechanistically distinct from dietary carbohydrate restriction. These include glycolytic inhibitors such as 3-bromopyruvate, which is more accurately described as a pyruvate analogue than a citrate analogue, and interventions involving mitochondrial or TCA-cycle-related metabolites such as citrate, acetate and alpha-ketoglutarate. Such strategies may influence cancer metabolism, redox state, epigenetic regulation or anaplerosis, but they do not directly establish that dietary carbohydrate restriction reduces tumour lactate flux. They should therefore be considered part of the broader metabolic-therapy strategy rather than evidence for or against the specific lactate-centred dietary hypothesis [90–94].

## Clinical Evidence and Research Gaps

### Limitations of Current Clinical Evidence

Clinical studies of carbohydrate restriction in cancer are still limited. Many are small, focus mainly on feasibility or safety, and do not measure lactate flux, immune profile, spatial microenvironmental changes or robust clinical endpoints [20]. Differences in dietary protocols, calorie intake, protein content, comparator diets and adherence monitoring make the evidence hard to interpret [74]. On present evidence, routine clinical use outside controlled research settings is not justified [67, 95]. To put the current trial landscape in context, registered interventional trials of caloric and/or carbohydrate manipulation in cancer have been summarised from ClinicalTrials.gov and International Standard Randomised Controlled Trial Number (ISRCTN) records accessed up to 28 May 2026. Eligible studies enrolled patients with cancer and explicitly manipulated dietary intake with the intention of modifying systemic metabolism. Trials were classified by the main dietary variable described in the registry record into caloric manipulation, carbohydrate-restrictive interventions, or mixed protocols. Classification was based on registry descriptions rather than reported outcomes.

The conclusion that carbohydrate-restrictive or ketogenic diets should remain investigational is not a rejection of their biological plausibility; it reflects the present clinical evidence base. Existing studies support feasibility in some settings and provide signals worthy of mechanistic testing, but they remain heterogeneous in tumour type, disease stage, diet composition, calorie/protein intake, adherence assessment, concurrent therapy and endpoints. They therefore, do not yet, support ketogenic or CRDs as anticancer treatment [20, 67, 77, 95].

While fuller details of each trial are provided in online supplementary Tables S1–S3 (for all online suppl. material, see https://doi.org/10.1159/mpp/adpag001), Table 1 summarises the number and scale of the trials with usable enrolment data. This overview shows an active but clearly still early-stage investigation. Across the 36 trials with verifiable enrolment figures, sample sizes ranged from approximately 10–602 participants, with an overall median of about 50. A few larger studies increased the mean, whereas most trials remained better suited to testing feasibility, safety, adherence and exploratory metabolic or immune endpoints rather than definitive anticancer efficacy. As the overall mean sample size of 96 is inflated by a small number of larger studies, the median of 50 better reflects the typical scale of the current trial landscape. Most importantly for the lactate-centred hypothesis, very few trials directly measure tumour lactate, lactate flux, lactate-related immune suppression or spatial metabolic changes within the tumour microenvironment.

**Table 1.** Summary of registered trials of caloric and/or carbohydrate manipulation in cancer

Online supplementary table/trial category	Trials with usable enrolment data, *n*	Approximate sample-size range, *n*	Mean sample size, *n*	Median sample size, *n*	Approximate total enrolment, *n*
S1: Caloric restriction/fasting/FMD/TRE	20	10–602	117	63	2,341
S2: Low-carbohydrate/ketogenic diets	14	12–170	50	41	702
S3: Mixed caloric and carbohydrate-restrictive interventions	2	40–356	198	198	396
Overall	36	10–602	96	50	3,439

Values are based on planned or reported enrolment in registered trials where a numerical enrolment figure was verifiable. Registry records were searched and verified through 28 May 2026. Fuller trial details are provided in online supplementary Tables S1–S3.

FMD, fasting-mimicking diet; TRE, time-restricted eating.

### Practical Limits of Large Dietary Trials

Large dietary trials in oncology are difficult in ways that drug trials usually are not. Baseline diets vary by culture, religion, income, family routines, cooking practices and long-standing eating habits. Cancer treatment can then change appetite, taste, gastrointestinal tolerance and even access to food. A “usual diet” or “control diet” is therefore rarely neutral or uniform. Complete dietary control is generally realistic only in short metabolic-ward studies, or in highly supported studies with meal provision. Those designs can be useful, but they limit scale, duration, external validity and equity. For large outpatient trials, the realistic aim is not perfect control. It is careful dietary specification, objective adherence monitoring, isocaloric and protein-matched targets where feasible, prespecified nutritional rescue, and sensitivity analyses that account for adherence, dietary displacement, and weight change. This is also why mechanistic endpoints matter. Without them, a null or positive clinical signal may be hard to attribute to carbohydrate restriction rather than calorie intake, protein intake, weight loss, cultural dietary pattern, or intensity of dietetic support [7, 76, 95].

### Future Trials Must Address Several Core Issues

These limitations lead to several trial-design requirements: clear dietary specification, control or measurement of calories and protein, objective adherence markers, lactate-related pharmacodynamic endpoints, biomarker-guided tumour selection, explicit handling of cultural and habitual dietary variation, and safety outcomes that capture body composition, function, quality of life and treatment tolerance [68, 96].

### Safety and Patient-Centred Outcomes

These should not be treated as secondary details. Weight, body composition, functional status, quality of life and treatment tolerance determine whether a dietary intervention is viable in cancer populations. Trial protocols should include stopping criteria for clinically meaningful weight loss or functional decline [80, 81, 97].

## Research Agenda and Trial-Design Considerations

### A Lactate-Centred Hypothesis Makes Concrete Predictions

A lactate-centred hypothesis makes testable predictions [29]. The intervention should measurably reduce lactate production, lactate export or lactate-dependent processes within tumours. Those changes should then correlate with biological or clinical benefit in defined contexts [59]. This distinction is important. Ketosis or dietary adherence can show that a patient followed the protocol, but they do not by themselves prove that the intended tumour biology was altered [68, 78].

### Trial Eligibility

Firstly, trial eligibility should be based on tumour biology as well as clinical characteristics [3]. The best candidates are patients with tumours that are strongly glycolytic, highly hypoxic and characterised by high lactate export [23, 29]. Histology alone will often be too crude, because metabolic phenotypes can vary widely within a single histological category [24]. A practical approach would be to enrich for glycolytic, lactate-high tumours using available methods: high FDG uptake on PET, high LDH expression in tumour tissue, high MCT4 expression suggesting lactate export, and hypoxia markers suggesting reliance on glycolysis [19, 60, 98]. Where possible, lactate imaging or metabolic profiling can give more direct evidence of lactate-rich biology [54, 55, 75]. These enrichment strategies should be specified in advance to avoid selecting “responders” after the fact [5].

### Dietary Intervention

Secondly, the dietary intervention must be described precisely enough to allow mechanistic interpretation [62]. The term “ketogenic diet” can cover very different macronutrient ratios, calorie targets and protein intakes. Carbohydrate content, protein intake, and caloric intake therefore need to be controlled or measured [26, 56, 74, 77]. If the question is carbohydrate restriction itself, an isocaloric design with fixed protein intake is essential. If the question is combined metabolic stress, any intended calorie deficit should be stated clearly [9]. In larger outpatient trials, investigators should also record baseline dietary pattern, cultural or religious dietary constraints, household food practices, food insecurity, and treatment-related symptoms because these factors shape both feasibility and the meaning of a “usual diet” comparator.

### Adherence

Thirdly, adherence should be assessed objectively, not simply assumed [53, 74]. Ketone measurements, such as blood beta-hydroxybutyrate or breath acetone, can confirm a physiological response to the diet. They do not prove that tumour lactate flux has fallen [54, 99]. Dietary logs, digital tracking, periodic recalls and, where feasible, meal provision can all help. Continuous weight and body-composition monitoring are also important because spontaneous hypophagia can easily confound interpretation [20, 53].

### Mechanistic Endpoints

Fourthly, mechanistic endpoints should be built into early phase trials as primary or co-primary outcomes [68, 96]. A lactate-centred framework points toward lactate-related pharmacodynamic measures [16]: tumour lactate concentration by magnetic resonance spectroscopy, lactate-to-pyruvate conversion by hyperpolarised imaging where available, or metabolomic proxies of lactate flux in tumour tissue and plasma [75, 100]. Immune and microenvironmental endpoints are also important. Changes in effector T-cell function, cytokine output, immune infiltration patterns, and myeloid suppressor populations could support the hypothesis that reducing lactate relieves immune suppression [35, 36]. Spatial profiling can then test whether immune changes occur in lactate-rich regions or at invasive margins, where lactate gradients may be most relevant [59, 101].

### Therapy-Combination Hypotheses

Fifth, therapy-combination hypotheses should be specified before the trial starts [66]. If carbohydrate restriction is being tested as an adjuvant, the study should be designed around the conventional therapy of interest [95]. In radiotherapy combinations, relevant endpoints include oxidative stress, DNA damage response and tumour control probability, ideally linked to lactate and redox measurements [69, 71]. In immunotherapy combinations, endpoints should include immune activation markers and functional assays integrated with tumour metabolic profiling [58]. Timing also matters. The dietary intervention may need to begin before therapy and overlap with it if the aim is to alter tumour biology during the relevant treatment window [9].

### Safety Monitoring

Sixth, safety monitoring should be designed for oncology patients, not borrowed uncritically from general dietary research [83]. Protocols should include minimum calorie and protein targets, dietetic support, body-composition assessment, and predefined stopping criteria for clinically meaningful weight loss or functional decline [8, 80, 81]. Treatment tolerance should also be recorded: dose reductions, delays, hospitalisations, and patient-reported outcomes [84, 97].

### Separating Biological Plausibility from Clinical Efficacy

Seventh, interpretation should keep biological plausibility separate from clinical efficacy [67, 78]. Early phase trials may show feasibility and may demonstrate that lactate-related biomarkers can be changed. That is useful, but surrogate changes should not be overread in the absence of meaningful tumour or patient benefit [96]. The main value of these trials may be to show whether lactate-dependent programmes can be modified in humans and to identify the settings in which larger efficacy trials are justified [75, 78].

### Ethical Considerations and Effective Communication

Dietary interventions in oncology sit in a difficult social environment. Patients are often exposed to strong claims and misinformation, including the familiar “sugar feeds cancer” message [12]. Trial teams and clinicians should be clear that carbohydrate restriction is investigational. It is not a substitute for standard therapy, and it can be harmful if nutritional status is compromised [102]. Protocols should guard against coercive or unrealistic dietary targets, respect patient autonomy and quality of life, and recognise that food choices are shaped by culture, family practice, finances and treatment symptoms, not just individual motivation [7, 96]. Finally, the lactate-centred approach should support integration rather than competition between diet and pharmacology. If dietary carbohydrate restriction reduces lactate flux in selected contexts, it could be tested alongside more direct lactate-targeted therapies, including LDH inhibitors, MCT inhibitors and interventions targeting pH regulation or lactate signalling [88, 89]. Concordant findings across dietary and pharmacological perturbations would strengthen causal inference.

Discordant findings would also be useful because they would help define the limits of upstream dietary modulation [76, 101]. Overall, the research agenda is clear: determine whether carbohydrate restriction can reproducibly alter lactate-dependent tumour biology in humans, and whether it can do so without compromising host resilience or treatment delivery [76, 95, 101, 102, 103].

## Implications for Clinical Practice and Policy

Until adequately powered trials with mechanistic endpoints are completed, CRDs should not be presented as anticancer therapies [56, 67]. Their possible role is as a supervised adjunct within research protocols or specialist services that can monitor nutritional status [7]. In counselling patients, clinicians should separate three questions: can the patient follow the diet while maintaining adequate intake; does the regimen risk weight loss, sarcopenia, metabolic disturbance, or treatment disruption; and has benefit been shown for that tumour type and treatment context [77, 102, 103].

Public cancer-nutrition guidance is primarily written to prevent malnutrition, support treatment tolerance and discourage unsafe dietary extremes. Those aims are important and should be preserved. However, when general healthy-eating models are transferred into cancer advice without explicit discussion of tumour glucose avidity and glucose-to-lactate metabolism, they may inadvertently imply that substantial starchy-carbohydrate intake is metabolically neutral in all cancer settings. The more precise message is not that patients should self-restrict carbohydrate, but that carbohydrate quality and quantity are biologically relevant variables. Public-facing guidance should distinguish nutritional support from tumour-metabolism claims, discourage carbohydrate excess without compromising energy and protein adequacy, and encourage patients interested in lower carbohydrate or lower glycaemic approaches to do so only with oncology and dietetic supervision. Representative examples of current public guidance are summarised in online supplementary Table S4. This communication issue is summarised schematically in Figure 2.

**Fig. 2.**
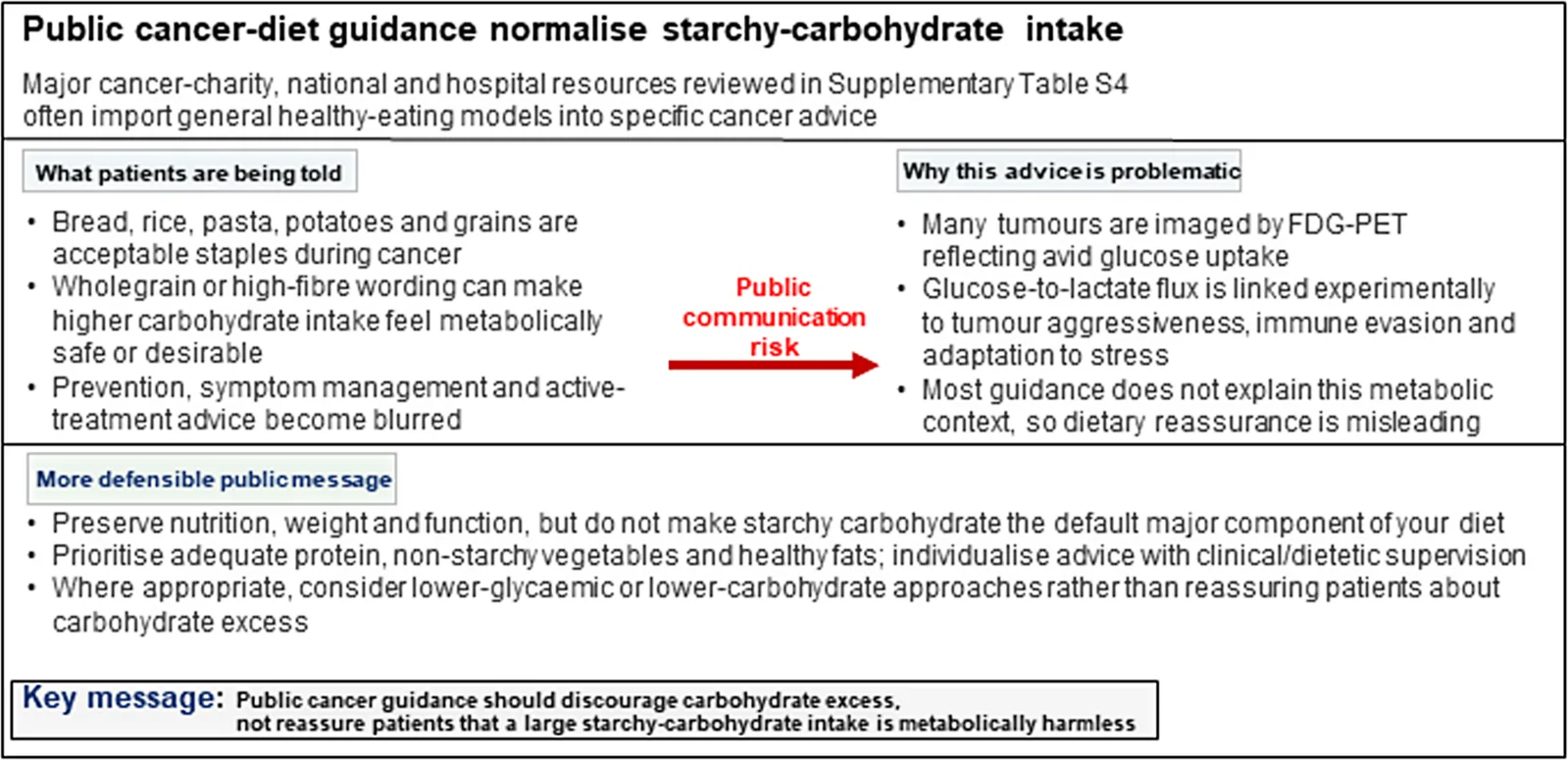
Public cancer-diet guidance and the communication risk around starchy-carbohydrate intake. Schematic summary of how patient-facing cancer-nutrition guidance may import general healthy-eating models into oncology without explicitly distinguishing supportive nutrition from tumour-metabolism claims. The figure contrasts the intended supportive-care purpose of such advice with the possibility that it may unintentionally reassure patients that substantial starchy-carbohydrate intake is metabolically neutral. A more balanced message would preserve energy intake, protein adequacy, body weight, muscle mass, and treatment tolerance while recognising that carbohydrate. Quality and quantity are biologically relevant and should be evaluated in supervised clinical studies rather than adopted through unsupervised self-restriction.

Health systems also need to consider implementation. Dietary interventions can widen inequity if they require intensive professional support, meal provision, digital tracking, or food costs that many patients cannot access. They can also create opportunity costs if they divert time and attention from established supportive care, such as optimising protein intake, treating cachexia-related symptoms and maintaining function during therapy. Research programmes should therefore include dietetic expertise, patient reported outcomes, and evaluation of cost and resource implications alongside biological endpoints [81, 96, 103]. A lactate-centred approach may help move the debate away from dietary opinion and towards testable mechanisms. It also encourages integration with pharmacological and radiotherapeutic strategies that target lactate biology more directly [16, 21, 71, 88, 89]. The most valuable contribution of carbohydrate-restriction research may be practical: to clarify when lactate dependence is clinically actionable and how lactate-driven microenvironments might be perturbed safely in humans.

## Limitations of This Review

This review is a narrative synthesis. It focuses on mechanistic plausibility and biological limits rather than providing a formal systematic review. It aims for conceptual clarity and trial-design guidance, not an exhaustive catalogue of every dietary protocol or outcome. The field is also moving quickly, and ongoing trials may refine or challenge some of the arguments made here. A further limitation is that diet cannot be standardised, blinded or controlled in the same way as a drug intervention. This makes trial feasibility part of the evidence problem, not simply a logistical issue. Even so, the lactate-centred framework provides a disciplined way to evaluate claims and design studies that can test them more directly.

## Conclusions

CRDs have attracted considerable interest as metabolic interventions in oncology. Their clinical relevance, however, remains unproven [67]. A lactate-centred view gives a more coherent rationale than the usual glucose-starvation narrative, but the relevant question is whether carbohydrate restriction can demonstrably weaken lactate-driven tumour ecosystems in defined settings without compromising host resilience [16–18, 101].

Current evidence is insufficient to support routine clinical use outside controlled research settings [20, 95]. The most useful next step is biomarker-informed clinical investigation that measures lactate flux, builds in nutritional safeguards and tests defined tumour types and therapy contexts [83, 99, 100].

## Acknowledgments

The author thanks the editors and reviewers for their constructive comments, which helped improve the clarity and balance of the manuscript.

## Statement of Ethics

Ethical approval was not required for this review because it did not involve new studies of human participants or animals. The manuscript is based on published literature and publicly available clinical trial registry information.

## Conflict of Interest Statement

The author declares no conflicts of interest.

## Funding Sources

No specific funding was received for the preparation of this manuscript.

## Author Contributions

Yunus A. Luqmani conceived the review, reviewed, and interpreted the literature and trial registry information, drafted the manuscript, revised it critically for intellectual content, and approved the final version for submission.

## Data Availability

No new primary data were generated for this review. The clinical trial information discussed in the manuscript was obtained from publicly available trial registry records, as described in the text and supplementary tables. Further details are available from the corresponding author on request.
